# The Term “Conservative Surgery” as It Has Been Proposed to Apply It to the Uterus and Its Appendages

**Published:** 1899-05

**Authors:** Lawson Tait

**Affiliations:** Birmingham, Eng., Late President of Mason’s College and Professor of Gynecology


### BUFFALO MEDICAL JOURNAL.

VOL. XXXVIII. MAY, 1899. NO. 10.

### Original Communications.

### THE TERM "CONSERVATIVE SURGERY" AS IT HAS BEEN PROPOSED TO APPLY IT TO THE UTERUS AND ITS APPENDAGES.

BY LAWSON TAIT, F. R. C. S. Eng., Etc.,
Birmingham, Eng.,
Late President of Mason's College and Professor of Gynecology.

I WAS very much amused some months ago by an article in the *Nineteenth Century* on Anesthetics, in which it was recommended to the laity that "each one of us should refuse resolutely to take chloroform or allow any member of our family to take it without first obtaining a guarantee from the anesthetist that he will administer it on an open cloth, held at a given distance from the nose, and that the time taken to put us under shall not be less than eight minutes." I was amused because the *Nineteenth Century* is published in London, the very hot-bed of new anesthetics and new methods of administering them. I was amused because here was Simpson, of 1851, coming back after nearly fifty years and his method being forced back on the medical profession through a lay journal—an offence of the direst description. The offence was resented, of course, by a large number of medical journals in this country, on the Continent and in America, and, of course, the greatest amount of fun was to be had out of the criticism and opposition of our trans-Atlantic friends.

One of the most prominent, and I think the best of the American medical weeklies had a leading article on the subject, in which the very difficult task was fully accomplished of admitting the need and justice of the outcry on the part of the patients as voiced by the *Nineteenth Century*. The fault in this country, says the article, is for the most part limited to the administration of ether, where the most inexcusable indifference to the patient's comfort is often demonstrated.

The man who gives the anesthetic in a hospital is regularly the youngest man on the staff, a fresh graduate with theoretic teachings alone, or the example of another interne, as his guide. The cone soaked with ether is crowded down over the patient's face and he makes a courageous effoit to stand it for a time. Very soon, however, he struggles to save himself from what these patients afterward describe as a feeling of imminent suffocation and death, and gains a breath of fresh air only in those cases in which the orderly or orderlies are unable to hold him down. It may be a little comfort that they are as bad in America in one way as we are here in another, but the whole thing is discreditable to our profession, and absolutely subversive of any claim ever to rank as a science. For here we have the greatest drug ever introduced, imperatively the greatest boon ever granted to humanity after opium, that is an ideal anesthetic to my mind, of course, chloroform; and we are not yet agreed about it, its method of administration, its power, and its method of fatality, in fact we are agreed only that it is an anesthetic. The history of this magnificent subject during the half century is a record of proceeding not only entirely unscientific, but eminently discreditable to a body of men who are supposed to have, by their examination in Euclid's six books, at least mastered the elements of the art and science of logic. It is a history of rash and unjustifiable introductions of new substances and new methods, going as far as the introduction of the old substance under a new name, "a substance much safer, more rapid, more and everything better than chloroform," by a man who was for a time a medical Cagliostro. We had, of course, hundreds of courses of experiments on animals in India and elsewhere, with conclusions as absolutely hostile as well could be. We have had initiated only one logical research in the shape of the "Anesthetic Committee," inaugurated by our own association, which still dawdles its slow length along and will report, as I am informed, about the year 1950.

If I had time I could illustrate my purpose as well in the matter of new drugs, but I shall content myself with one short quotation concerning one of the newly passing foibles, "Thyroid" and its use in bleeding fibroids. In the *Medical News*, New York, page 17, Moseley reports "that while some patients can take comparatively large doses of thyroid with impunity, others are injuriously affected by small amounts," and having established this extraordinary conclusion concerning our cherished sweet-bread, he tells us that they have a marked influence in bleeding fibroids, in checking the excessive loss of blood, and in some cases in diminishing the size of the growth. His observations were extended over five cases. and is an example of the sort of thing which almost renders our desire to see new drugs kept out of the market by act of parliament till they had passed through a mill of judicious and stipendary investigation.

The fault of this logical deficiency is not, of course, confined to affairs medical, for we find the general affairs of the whole nation robbed, say at a general election, by the words and votes of the floating residum of voters in the most important, and generally therefore the most incompetent electoral country, and we seem now to be on the threshold of a vast upheaval within the serene demesne of our National Church, the result of a ruthless spirit of innovation and desire for new methods and new measures, not even a revival of the good old things so faithfully followed by our ancestors for over a thousand years.

But in matters medical we ought to do better than that, particularly is it possible in matters of surgery when we have proof of results not tangible in the sister art of medicine, save within very limited lines.

For the last forty years, I think, hardly an inaugural address dealing with the generalities of the advances of surgery has omitted reference to the history of ovariotomy as one of its crowning glories. But I do not accept the story in that way at all. I think the whole thing discreditable, and for one reason only, even if no others were available, that whereas Nathan Smith penetrated the secret to the very depth, and published it in 1827, his great discovery was pushed aside by "new methods," and the great advantage of it was withheld from suffering humanity for more than half a century. Surgical historians, writing towards the end of the coming century, will not look on this as a brilliant record.

Arrived at the year 1878 we put the removals of ovarian tumors down with a mortality of 5 per cent. and then paused to look around. The start of abdominal surgery from this point was made when I showed that 100 consecutive exploratory incisions could be done with a hardly appreciable mortality, and then I formulated and established the law that when conditions in the abdomen threatened life or made it unbearable, we were justified in opening the abdomen to discover the site and nature of the disease. Out of this, as a matter of course, arose one by one and rapidly, as my records show, all the modern and fully accepted operations for gall-stone, etc., which now fill large text-books. Among these records fully numerous operations on the uterus and its appendages, most of which have become classic, and will remain so, unless upset for a time by men more restless and new methods wholly unnecessary.

It is needless to say that in this losing battle, going over now a period, so far as I am concerned of more than thirty years, I have not, I did not consider it necessary, and it certainly would not have been advisable to make public every step in the process. Mistakes were made, methods employed were found to be faulty and unsuccessful and nothing would have been gained by dilating upon them or even drawing attention to them. Just as I started, Dieulafoy had introduced his aspirator—an instrument in the device of which he had been anticipated by Bowditch and Protheroe Smith; indeed by many others, until we get back to the Roman surgery of Pompeii; and Maisonneuve had fought the battle of the drainage-tube. The pelvis did not escape either of those proceedings and they were fully employed. Between 1871 and 1878 my carriage bag had always had an aspirator, but for at least twelve years I have not used it. With it I carried a number of ingenious devices for making openings in the vagina, and getting drainage-tubes into something, never knowing exactly what, sometimes curing, but more often failing to do real permanent good. But as it dawned on me that the peritoneum had no real terrors if respectfully treated, I found that it was better to ascertain accurately what was needed by careful ante-mortem examination, rather than make hap-hazard shots from below, and after a series of trials came my paper on Treatment of pelvic suppuration by abdominal section, which revolutionised the practice of pelvic surgery all over the world. Not only so, but it cleared up the pathology of the pelvis and put a stop to the eternal and ignorant wrangling about perimetritis and parametritis, which had gone on for nearly thirty years. Of this, the present generation has no need to know anything, and truly it does not, as may be seen from the contents of a paper by Dr. Noble, in the *Philadelphia Medical Journal*, of July last, entitled the Conservative treatment of pelvic suppuration of puerperal origin. He begins, as is not unusual with such people, by reference to, and conclusions derived, according to his interpretation from my own writings, and succeeds, as is equally usual, in completely distorting my teaching. He goes so far as to quote my own words almost verbatim and then commenting on them in this sentence: "These elementary pathological facts are now generally recognised, although some years ago they were sharply controverted, more especially by the disciples of Tait."

According to his contention the truth was first established by the publication of four cases of True pelvic abscess, by Dr. Charles P. Noble, in August, 1891, before English gynecology was born. He gives his idea of the differential diagnosis between pelvic cellulitis and pus tubes or intraperitoneal abscess (regarding these two as the same) and misses the fundamental parts of my teaching on this subject, first published ten years before his paper of 1891, that there are two varieties of intraligamatous suppuration, one in the outer half of the layers, which he has recognised and another in the inner half, quite as easy to recognise, is found in the left side, having a sign which there is no mistaking, and then he goes on to describe such a case which he did not diagnosticate, but mistook when he need not have done; for which he opened the abdomen correctly enough but did not proceed to complete the operation as he should have done, and subsequently attacking the abscess from the vagina, as he might have done at first if he had been at all familiar with my teaching, or had been one of those disciples upon whom he pours the vials of his contempt.

It is not for such blundering as this that I now draw attention to Dr. Noble, but because he is an example, though a very bad one, of the clouding school of abdominal surgery. Of these, most exist in Germany, and the treatment seems to be reaching England, and whilst I would be the last, by my utmost endeavor, to deride change merely because it was new, I deprecate change when introduced for no other purpose than its novelty, when its novelty leads us off the track of the investigation of facts already quite familiar to us, but concerning which we have not arrived at final conclusions.

That suppuration in the plevis should not be made the subject of surgical rule different from that affecting other regions had to be my cry for nearly ten years, or as I put it that a surgical crisis shall rise in the pelvis as it does in the knee-joint, and I carried my point against all comers. The chief opposition was, of course, from the uterine tinkerers who were overwhelmed by seeing their consulting rooms emptied of the helpless sufferers, who came day after day for a glycerine plug, or month after month for new pessaries. They raised the cry of spaying women, emasculation of women (a strange mixture of etymological definition) and even went so far as to say that we surgeons operated for the fees at the end of the cases. This cry ceased, however, when it was shown that the operating table of the surgeon was much cheaper in the long run than the consulting room of the pessary-monger; and, personally, I am now in a position to say that I should have been a much richer man if I had never seen any of these cases, for as I had to give bed-room to the great bulk of them I was out of pocket, and largely, by the whole transaction. The strange thing is that the cry is still kept up by beardless boys who tells us what they can have no personal knowledge of, whether it be true or not "that the reckless way in which, in the past, gynecologists have removed uterine appendages without adequate justification, is the opprobrium of our art. In the past I was in the habit of checking such nonsense by saying that such informers either knew of such proceeding or they did not. If they did not they were liars. If they did it was their business to give such information as would put the offenders on their trial for felony, and failing to give such information, they were accessories after the fact and so liable to indictment themselves. I went so far as to serve notice on a well known London physician who was guilty of this, that I should prosecute him if he did it again, and since that time he has been quiet.

Another parrot cry still being repeated was that most operations made the patients absolutely sterile. But then they were so rendered by the disease before our operation. Disease of the lower joint makes a man lame, amputation of the limb confirms the lameness, and the best case of a "conservative" result of an excised knee-joint I have ever seen did not get about free from lameness—in fact, I think the lameness was worse than it would have been with a first-class artificial limb. Double pyosalpinx renders a woman absolutely sterile. Nay, more; as a rule, she cannot have intercourse. Removal of the appendages does neither increase nor decrease the sterility and it often removes completely and permanently a grave interference with marital life. Opening and draining a double pyosalpinx may do as much, but that it will cure sterility—pigs may fly, but I have yet to see a flight of them.

Foiled in all such arguments, our critics have found another platform, and I take again Dr. Noble as my example—he is as good for my purpose as any other of the score from which I might select, they are all as deficient in logical acumen as he is, they put their cases quite as badly, and all are as open to the same suspicion of quackery; there is no use mincing terms or using plausible phrases to hide the pill which must be swallowed.

It is forgotten, or at least seldom acknowledged by gynecologists, that the adoption of measures does not rest with them, but with the mass of the profession, men in general practice, upon whom rests the responsibility of advising their patients for what seems to be the best. It is perfectly true that the profession will be and must always be governed by the weight of a great name, so that I had a very hard fight after the International Congress, at London, in 1881, when Spencer Wells said that he had only once seen such a case as I described in his life. I answered by publishing a long series of my own cases which had previously been under his own care. This cost me his personal friendship, a result I regretted as long as he lived, and still regret, but I won the battle. I silenced Matthews Duncan by compelling him, almost by threats, to come with me to a neighboring house in London, to see me remove two huge bags of pus in a patient under the care of Dr. Chapman Grigg, which had quite recently been under that of Dr. Duncan. I met the personal difficulty with another case in London, when six distinguished practitioners met me and assured themselves that I had exaggerated my diagnosis, that there was nothing much the matter, by taking Ruth to London, making him operate, and then submitting to them further report of the operation.

Now another battle rages, and I shall halt no more in it than I did in the former, though my fighting powers are no longer as sharp as they were nor my taste for warfare as great. We are told again, though the contrary has been proved over and over again, that in a considerable majority of cases there is a diminution or total abolition of the sexual instincts. This is not true, in fact it is absolutely untrue. It is a subject on which, of course, the publication of facts is extremely difficult, either one way or the other. But my own facts establish the conclusion that the cases of abolition are extremely few, not more than 5 per cent., but they get greatly tattling about by loose-minded women and by men whose senses of honor and proper reticence in matters concerning their wives is strangely defective. On the other hand, the instances of restitution of marital relations, which had been entirely destroyed by disease and restored by the operation required are at least 60 per cent. of all the cases. In a few instances the mysterious fact remains that women who before operation had little or no sexual appetite, had it developed after treatment to an extent which becomes inconvenient. I removed the appendages, twelve years ago, of a lady noted in public estimation to the highest degree. She had had one child, and her husband had never shown any sexual response whatever, till the operation, when it became oppressive to him and he died. She lived as a widow for three years, applying to me from time to time for arrest of this symptom, until it got so bad that I advised removal of the uterus, and this I carried out not only without benefit, but rather with an increase of the trouble. She greatly objected to the idea of a second marriage and had always resented my advice, and the advice of her parents, in that direction; but at last, and entirely to save her conscience from the reproach of wrong, she married again, and a few months ago the fact was announced in every paper in Europe. It is therefore perfectly useless to say that in a few cases the sexual tastes are destroyed. What kind of an argument is it, I shall consider after ward, but meantime I go on discussing it on what merits it has; to prove the argument, and put in semi-decent form, the word "conservative" is introduced, a piece of rampant deceit and chicanery.

Dr. Noble has two papers that I have already quoted, and another on The conservative treatment of fibroid tumors by myomectomy. The paper is hardly worth referring to, reeking as it does of all the work of Simpson, Pean, Marion Sims and others of less note, who brought myomectomy as far as it would go, more than a quarter of a century ago, and thus it was left by all of us. But the paper is a useful warning of a common logical error, the use of the one distributing middle-term, or in plain words, the use of a term devoid of definition and employed with various and irreconcilable warnings in the course of the same argument. Thus in the one paper he alludes to the preservation of parts of ovaries diseased and adherent, and bits of occluded tubes as "conservative surgery," whilst in the other the same thing means removal of the whole uterus, the absurdly so-called pan-hysterectomy, revived from the moral of Pean's morcellement and hashed up as something new.

The term "conservative surgery" was introduced by Fergusson in the late "fifties," and was the cause of a hot feud between him and the more logically-minded Syme. But, as Fergusson puts it, it was fair enough, and became afterward limited almost entirely to its best example, that of excision of the knee-joint. Fergusson was a prince of operating surgeons, and none who can remember his magnificent figure and wondrous success, unmoved as he made the knife fly at things in the old days, with a rapidity which few eyes could adequately follow, can easily believe that such an operation as excision of the knee-joint would meet with his strong approval. In theory it was all right. Conserve the lower limb; it must always be better than an artificial one. But, alas! though his pupils carried the banner of this conservatism far and wide, it did not keep its promise, and on Syme's face there was to be seen, and there only, that strange little smile of triumph as he lopped off a "conserved"

leg." "Conserve the parts," was his only comment, and the operation took a back seat and occupies now a very restricted area.

Fancy my astonishment, therefore, when I read in the second paragraph of Dr. Noble's second paper this sentence: "In the recent past, true conservatism—that is, the welfare of the patient—has required," etc. This is another theory, and completely confirms my life-long belief in politics that the advanced Liberal is the truest Conservative. Certainly, with this definition, we all are, all hope to be, all must strive to be truly conservative. But why should Dr. Noble pretend to be that, and exclude others equally earnest in their efforts, and use for himself a phrase which reminds one of the story of the two men who went up into the temple to pray. If he claims exclusively to be a conservative, then I say he is also a Pharisee, as are also those who use this phrase as he uses it.

For what is its real intention, other than to declare that he has, and uses, a method of operating which conserves the sexual instinct, as he plainly does in his concluding sentence, together with a hope that the sterility may not be complete, nor completed.

In all such cases the probability of the restoration of fertility is so minute in its uses that it is wholly unworthy of discussion or acceptance, since in some very unusual set of conditions, as when the succession to a crown is concerned, and then, I, for one, would not accept the responsibility. Crowned persons are a risk unto themselves, and I never cared to share in the government. For the general public the terrible facts of over-population, and the fact that of all children born one half died before they are five years, was enough to dismiss such a bagatelle from my mind. When such patients have been married for years and remained sterile, it may be assumed that the sterility is complete and when the mischief has resulted after the birth of one or more children the patients themselves usually dismiss the argument with impatience. The second question, I find, is now being directed towards the apprehension of the husband more than to his partner, and here is where the quackery comes in.

In a recent case where I was concerned, and where it was strongly wished that the case should be got out of my hands, it was urged that whether on the one hand I should geld his wife and use, like a log of wood another surgeon, strongly recommended as of the conservative school, would leave her active, the husband promptly replied that the case was that of his wife not that of a strumpet, and the efforts failed. I confess that this is how I should look at the question, especially in the light of the public experience of Landau[1] and others who are practically the leaders of this new movement, aided by what is probably of far more importance, my own wish and longer established experience. The whole thing is based on a misconception of the function of the ovaries, which have no more to do with the sexual appetite than the kidneys. Nor have the fallopian tubes, nor the uterus, as it is maintained, and as I have proved, sometimes increased by the complete absence of all five. The two most erotic women I have ever come across were two sisters in whom not the slightest trace of uterus and ovaries could be determined, and in one of them I had positive knowledge of the fact of that complete absence when I operated upon her for tuberculous peritonitis. They were in good social position, were not insane, yet no kind of inducement, social, parental or restrictive could prevail upon these women to refrain from inspiring every man with whom they could get an opportunity to have intercourse with them, and *faute de mieux*, they were confirmed Sapphists as well as the daughters of a well-known physician.

Finally, is it an argument, even if it could be sustained, which we as surgeons, can give weight to?

I do not often quote Scripture, but I think that a great clinical lesson on this subject may be got in the V. Chapter of Matthew, when at the 29th verse we are told that "if thy right eye offend thee, pluck it out, and cast it from thee. And if thy right hand offend thee, cut it off and cast it from thee; for it is profitable for thee that one of thy members should perish, and not that thy whole body should be" infected with bacilli, a lesson which is still more emphatically localised as pertinent to the present man by my national poet in words which read:

> "Geld you, quo he, and what for no?
> If that your right hand, leg or toe,
> Should ever prove your spiritual foe
> You should remember
> To cut it off—and what for no
> Your dearest member?"

We are now called upon to play the part of moralists, but this we are called upon to do, to adopt as our guiding principle to do the best we can for our patients and for our art, and then for ourselves. I have always held in detestation any surgical proceeding which brings with it a risk that the patient shall have to submit to a second operation. I have, therefore, always steadily opposed all operations for malignant disease undoubtedly pronounced. Nothing brings so

1. We are not quite sure as to this name. It is Laudson in the copy, but we think Landau is intended.—EDITOR.

much discredit on our art, nothing so much discredits the individual practitioner. Not only so, but second operations in the pelvis are always more difficult than the first, and according to all published facts far more fatal than operations completed at first. So much is this the case that I have been driven by the stern logic of facts to advise a more complete operation than ever in certain kinds of disease of the appendages. I refer to those in very young women, when the mischief has arisen apparently at, or even before, the molimenal period. I am certain that of all the cases of suffering from the results of chronic inflammation of the uterine appendages they suffer the most, their sufferings begin soonest, and they are the most difficult to relieve. Among them I have had my worst and most bitter disappointments of relief expected, and have had discredit in this class of cases. About eight years ago I removed the uterus in one young lady, from whom I had removed the appendages, thirteen years before, not only with a failure as a result, but I had made her worse; she had taken to drinking, been placed in an asylum and had altogether gone to the bad. I removed her uterus and cured her promptly and, after nearly eight years' success, I think I may say permanently. I followed this case up, and hunted up a number of my known failures, removed the uterus in eleven more cases and have succeeded in all and I am now hunting up some more. This is conservative surgery. As a cross to it, I was called to London a few weeks ago to a consultation with one of the eminent west-end doctors on a case of this kind. I advised a complete removal, but the patient was conservative in the other way. She was sure that it was all in the right ovary, and the right ovary alone was to be removed. After much arguing and a full understanding in black and white, I consented to divide the operation and removed the right ovary and tube. We shall see. I do not usually accept a fettered condition such as this, but my colleague is a man for whom I have a great reverence and he took the responsibility, therefore I acquiesced.

My belief is consummate in such radical surgery as will preserve my patients from further risk, and I do not regard the "sexual appetite argument" as worthy of any but the brothel-keeper with whom it would, of course, have great weight. Concerning the surgical difficulties and dangers of the so-called "conservative" operation, my old experience of the early "seventies" are confirmed in a paper by Mr. Stanmore Bishop, in the *Medical Press and Circular* for November 23, 1898, and I need do no more than refer to that common-sense contribution to the subject.

